# Differences in trophic resources and niches of two juvenile predatory species in three Pangani estuarine zones, Tanzania: stomach contents and stable isotope approaches

**DOI:** 10.1186/s40709-018-0084-4

**Published:** 2018-07-03

**Authors:** Alistidia Paul Mwijage, Daniel Abel Shilla, John Ferdinand Machiwa

**Affiliations:** 1 Tanzania Fisheries Research Institute (TAFIRI)—Kyela Centre, P. O. Box 98, Mbeya, Tanzania; 2 College of Agricultural Sciences and Fisheries Technology, University of Dar es Salaam, P.O. Box 35064, Dar es Salaam, Tanzania

**Keywords:** Juvenile fish, Predator, Trophic resources, Niches, Pangani estuary

## Abstract

**Background:**

Estuaries are primary habitats that serve as feeding and nursery grounds for most juvenile marine fish. However, estuaries have been used as fishing grounds by the artisanal fishers in Tanzania. The slow-growing predatory fish at juvenile and sub-adult stages are among the most frequently caught species that functionally enhance multiple linkages of energy pathways within the food web. Stomach contents and stable isotopes (δ^13^C and δ^15^N) were used to describe the nutritional sources and trophic niches between the co-existing benthic, predatory species, *Carangoides chrysophrys* and *Epinephelus malabaricus* in the Pangani estuary, Tanzania.

**Results:**

The findings indicated significant inter-specific variations in dietary composition (PERMANOVA, *p* = 0.001, pseudo-*F* = 15.81). The prey-specific index of relative importance (%PSIRI) indicated that juvenile shrimps (%PSIRI = 51.4) and Teleostei (%PSIRI = 26.5) were the main diets of *C. chrysophrys* while brachyura (%PSIRI = 38.8), juvenile shrimps (%PSIRI = 25.6) and Teleostei (%PSIRI = 23.3) were important diets of *E. malabaricus*. The isotope mixing models indicated that the predatory fish species accumulate nutrients derived from similar autotrophic sources, microphytobenthos, seagrass and macro-algae via consumption of small fish, including clupeids and mugilids. Yet, they significantly showed different isotopic niche width with varying degree of niche overlap across the longitudinal estuary gradient. This situation was justified by the presence of basal food sources among the estuarine zones that isotopically were different.

**Conclusion:**

The reliance of both predators on clupeids and mugilid preys that are trophically linked with estuarine and marine basal food sources, is an indication of low estuarine food webs’ connectivity to the fresh water related food web. This situation is most likely threatening the stability of the estuarine food web structure. Management strategies and plans in place should be cautiously implemented to ensure the balanced anthropogenic freshwater use in the catchment and fishing activities, for the maintenance of the Pangani estuarine ecosystem health.

## Background

The stability of the food web structure is one of the main determinants of an ecosystem’s health [1]. Preservation of estuarine food web structure is thus vital for maintaining the ecological and functional roles of an estuarine ecosystem. Estuaries in the world are primary habitats for the growth and survival of most juvenile marine fish [2, 3]. They serve as nursery grounds since they provide diverse food and shelter for most marine fish across all trophic levels along the estuarine food web [4, 5]. Such fish community assemblages comprise predatory fish which are economically and ecologically important in the mangrove estuaries along the coast of Tanzania. In these habitats, artisanal fishers tend to fish unselectively by using inappropriate fishing gears [6]. This is a concern especially when the species caught include slow-growing fish that attain large body size before reaching maturity [7, 8].

The dominance of many marine juvenile fish in the Pangani mangrove estuary along with intense fishing practices raises concern about the abundance of resource base and how do predatory fish species with similar feeding habits, partition dietary resources and thus, manage to co-exist. The presence of species that occupy the same trophic level and demonstrate similar trophic niches in such a highly disturbed estuary can be an indication of the stability of the food web structure [6]. This is frequently related to the occurrence of predatory fish with trophic linkages originating from diverse trophic base resources [9, 10]. Under such circumstances, loss of connectivity within and across trophic levels is unlikely to occur due to energy flow higher up the food web through multiple channels [6, 10]. Pelagic, benthopelagic and even benthic predatory fish are important to maintain the food web stability since they link pelagic and benthic food chains in aquatic ecosystems through feeding relationships [10, 11]. In addition, knowledge on the trophic interaction among autotrophs, primary consumers and predatory fish can help describe how these predators affect and depress the biomass of organisms down the food web. Studies on the trophic relationships of marine juvenile predators that link top predators and primary consumers are limited in estuarine systems [10, 12] particularly in Tanzania where estuaries are highly disturbed due to fishing.

Stable isotopes and stomach content analyses are methods that complement each other to gain more insight into the feeding relationships of fish species and the degree of dietary resources sharing in different aquatic systems [2, 6, 13, 14]. Integrating these two methods is important for the interpretation of feeding results. Through stomach content analysis, it is easier to determine what types of prey (at high taxonomic level) are instantly ingested by predators [15]. However, the method does not allow the assessment of what types of prey have been historically assimilated by the species which is revealed by stable isotopes [16]. Carbon and nitrogen isotope ratios reflect long-term diets [16] and quantify the primary food sources [17] assimilated by the species during the time the organism is growing. The use of stable isotopes is important, especially when characterizing the diet and feeding interaction of predatory fish species like groupers that have high prey regurgitation and stomach vacuity index [18]. Due to the ability to integrate assimilated dietary signatures on a long-term basis and differentiate dietary sources of marine and fresh water origin, stable isotope analyses can better infer the nutritional sources and feeding relationships of juvenile marine predatory species throughout the period they spent in the estuarine nursery ground.

Juvenile marine fish are currently dominating fish assemblages in the Pangani estuarine system. This has resulted by the increasingly inland intrusion of sea water through tidal actions as a consequence of reduction in fresh water inflow into the estuary [19–21]. The predatory marine fish species present in the estuary among others, include lutjanidae (e.g. *Lutjanus argentimaculatus*), sphyraenidae (e.g. *Sphyraena jello*), hemiramphidae (e.g. *Hyporhamphus dussumieri*), gobiidae, serranidae (e.g. *Epinephelus* species), carangidae (e.g. *Carangoides* species) and others [22]. These species differ in salinity tolerance along the salinity gradient, as well as feeding habitats and area within the water column [23]. The feeding of some fish species is directly or indirectly connected with the mangrove habitats. Few marine predatory species are abundant in the upper Pangani estuarine zone where the banks of the estuary are fringed with coconut palm trees. Lower and middle estuarine zones are dominated by mangrove forest [24]. Greasy grouper or black-spot estuary cod, *Epinephelus malabaricus* and longnose trevally, *Carangoides chrysophrys,* are abundant predator species in estuaries at juvenile and sub-adult stages [7, 23, 25, 26]. In the Pangani estuary, they are abundant in the lower, middle and upper or innermost zone from the estuary mouth. They are among the multiple fish caught by local fishermen throughout the Pangani estuarine zone. Unfortunately, no documented data are available for the movement, feeding pattern and other ecological features of these species in Tanzanian estuaries [8]. A study conducted by Bhat et al. [23] in the Aghanashini estuary, in India indicated that *C. chrysophrys* was abundant throughout the year and across the estuarine reaches with average salinity ranging from 6.5 ppt (inner estuary) to 22.5 ppt (near estuary mouth).

Like other groupers which are slow growing, juvenile *E. malabaricus* use estuaries and other shallow habitats over a year and after reaching sexual maturity they migrate to deeper waters for spawning [7]. This grouper is a protogynous hermaphrodite [8]. Therefore, uncontrolled fishing of pre-mature individuals in the Pangani estuary can interfere with the reproduction cycle of the species. This grouper is also a voracious predator, feeding on fish, macro-crustaceans and in some cases even on octopuses. The species uses a sit and wait, ambush hunting style foraging behavior, and it is tolerant to low salinity and turbid environment [27]. The species is in the list of near-threatened species as mentioned in the International Union for Conservation of Nature (IUCN) Red List [7]. Likewise, *C. chrysophrys*, despite being a fast swimmer roving in nature, juvenile and sub-adults tend to spend a certain period of their time in estuaries and other coastal shallow water habitats and move out as they grow [25, 28]. Males of *C. chrysophrys* attain sexual maturity at 46.90 cm in length and females at 42.08 cm [26]. The duration of stay of *C. chrysophrys* in the estuaries is not well known but literature indicates that it takes more than 2 years to reach maturity [25, 26]. Previous studies show that both species can compete for the dietary resources in estuaries as they consume small fish and epibenthic crustaceans including shrimps and crabs [25, 26, 28–31]. The high artisanal fishing pressure in the Pangani estuary with multiple catches including all available predatory fish species poses a need to identify the trophic niches and relationships of *C. chrysophrys* and *E. malabaricus*. These two species share trophic resources and feeding grounds, in particular, in the Pangani shallow estuarine system.

Therefore, the present study aimed at characterizing the trophic relationships between the juvenile co-existing predatory species, *C. chrysophrys* and *E. malabaricus* throughout the Pangani estuarine system by using stomach contents and stable isotopes of carbon and nitrogen. Specifically, the study focused on: (1) describing the main dietary composition in these species and their level of variation (2) assessing their sources of nutrition in a longitudinal salinity gradient in order to better explain their level of similarity/differences in trophic niches, (3) assessing their isotopic niches. The study answered the following questions: To what extent do the diets of these two species differ along the longitudinal estuarine salinity gradient? Do their sources of nutrition differ and to what extent? How do their trophic niches differ? Answering these questions will highlight the degree of the species connectivity to various trophic bases and resource partitioning, which is essential information when evaluating the estuarine community structure over time.

## Methods

### Study area

**Fig. 1:**
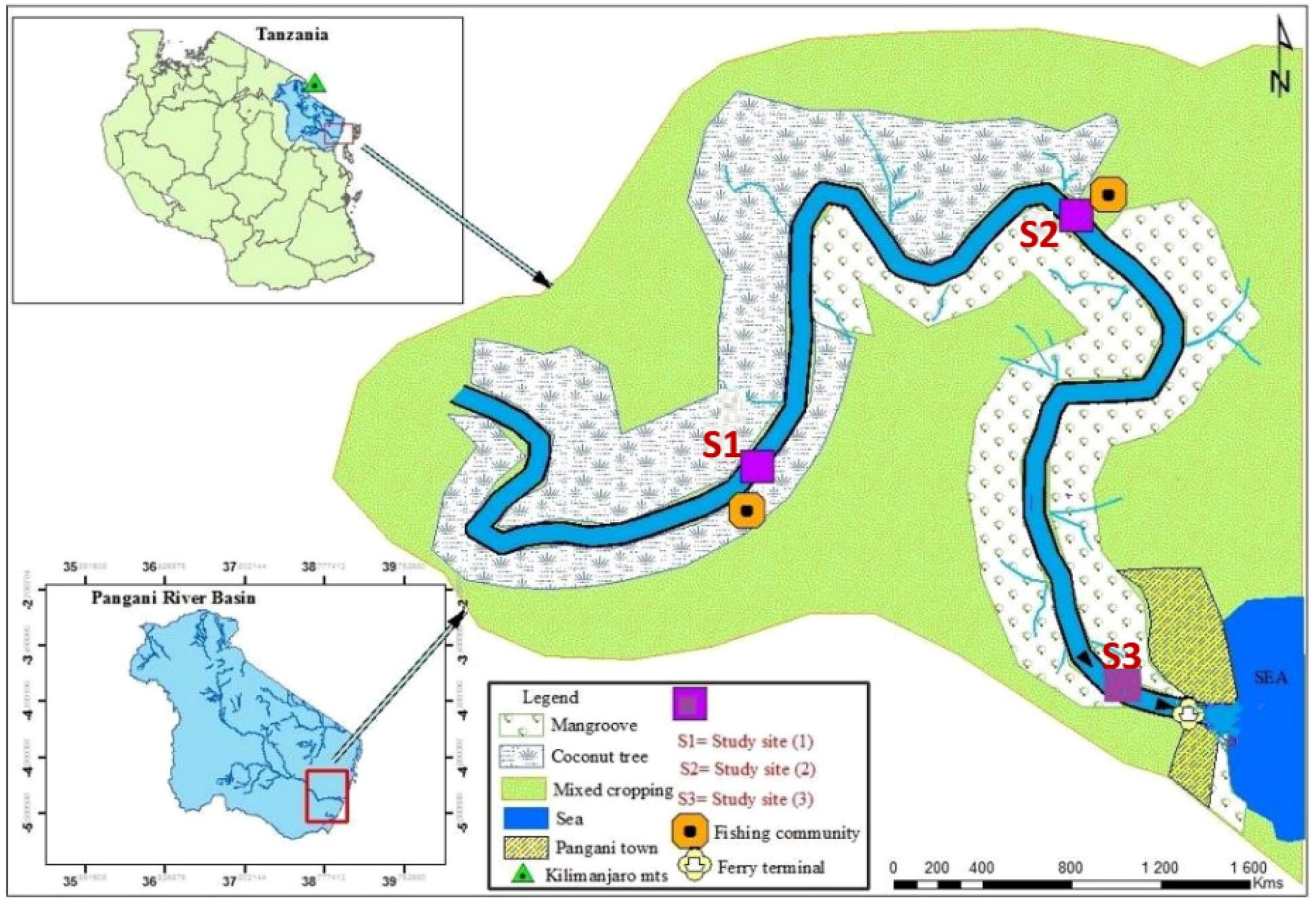
Map of the the Pangani estuary showing the sampling zones. The site 1, site 2 and site 3 marks indicate the end of the upper, middle and lower sampling estuarine zones, respectively

The study was carried out in the Pangani estuary located along the west Indian Ocean, in northern Tanzania coastal areas at 38° 50′E, 5° 20′S and 39°E, 5°26′S [32] (Fig. 1). The estuary is permanently open, funnel-shaped and part of the Pangani River Basin of about 43,650 km^2^ [19, 33]. This basin is the third largest in Tanzania. Pangani is a macro-tidal estuary of semi-diurnal type with an amplitude of about 3.5 m during the spring tides and 3.0 m at neap tides [19, 34]. The estuary has an average depth of 5 m and is very turbid in its upper and middle reaches [19]. It is characterized by eroded banks, extensive mangrove forest interspaced with coconut trees and clay and silt mud flats. The fresh water discharge into the estuary has decreased as a result of water abstraction upstream of the river catchment [19, 20, 33]. Due to low river discharge and high tidal range, the estuary experiences strong mixing of fresh and saline water. Samplings were carried out in thee sampling zones located along the longitudinal salinity gradient from the estuary mouth, in order to account for the foraging movement pattern of marine predatory fish species in the estuarine feeding grounds. The lower estuary extended from the estuary mouth to 3 km upstream followed by the middle estuary from 8 to 10 km and, the upper estuary at about 14 to16 km from the estuary mouth (Fig. 1). The average salinity was 5‰ in the upper estuarine zone, 14‰ in the middle zone and 30‰ in the lower reaches of the estuary.

### Field sample collection

Two predatory marine fish, *C. chrysophrys* and *E. malabaricus* and their potential preys and autotrophic sources were collected separately in the three sampling zones. Predatory fish and potential prey fish were caught by using seine net and monofilament gill nets of multiple mesh sizes at spring tides in October 2014, January, March, May and July 2015. The prey fish caught were dominated by clupeids (*Hilsa kelee*) and mugilids (*Valamugil buchanani*). After retrieval of the nets, prey fish and various sizes of the targeted predatory species, were selected and stored in a cool box with dry ice. Later on, wet weight (to the nearest 0.1 g) and total length (to the nearest 0.1 cm) were recorded, and predatory fish stomachs were removed and labeled. All fish samples and stomachs were frozen at − 20 °C on the same day. Overall, 135 samples of *C. chrysophrys* and 164 samples of *E. malabaricus* were collected and preserved for stomach contents analysis. For the prey fish, 64 samples of Mugilids and 59 samples of Clupeids were preserved for stable isotope analysis.

Along with fish sampling potential prey, invertebrates and autotrophs, were collected for stable isotope analysis. The fiddler and swimming crabs, gastropods and bivalves were collected by hand in the intertidal areas during low tides. Shrimps, dominated by *Fenneropenaeus indicus* and palaemonidae, *Sesarma* species and *Scylla serrata* crabs were obtained from fishermen at the time of retrieving their nets in each zone. Isopods were collected from barnacle shells. Amphipods were collected by a hand corer in shallow waters. The sediment cores were sieved through a 250 μm sieve, and the retained organisms were sorted, collected into containers and stored in a cool box. Three replicates of surface sediment were collected in shallow waters by using a cylindrical hand corer and the top 5 cm layer was removed and frozen for isotope analysis of surface particulate organic matter (sPOM). Microphytobenthos were collected by taking the top surface sediment layer (1 cm), using a hand corer in the unvegetated intertidal area, which was mixed with distilled water and filtered through a 63 µm sieve. The supernatant was incubated with pre-treated sand (acid washed and combusted) and then illuminated for 7 h, a process that facilitates microalgae to be phototactically attracted to the surface top layer of the sand. Separation of microphytobenthos from sand was done through treating the mixture with distilled water, and filtering again through a 63 µm sieve and a GF/F glass fibre [35]. Samples of periphyton were collected by scraping rocks and fallen trees using a metal spatula. Macro-algae (green filamentous algae and *Sargassum* species) and sea grasses were also collected by hand. Leaves of mangrove trees *Avicennia marina*, *Rhizophora mucronata*, *Bruguiera gymnorrhiza*, *Ceriops tagal*, *Sonneratia alba*, *Heritiera littoralis* and *Xylocarpus granatum* were picked from tree branches only in the middle and lower estuarine zones; they were absent in the upper zone. Angiosperm (C_3_ and C_4_) plants including water hyacinth samples, palm leaves and Poaceae family grasses were picked by hand in the intertidal zone of the upper and middle estuary zones. All specimens after collection were bagged separately, stored in a cool box with dry ice in the field and later preserved in a freezer at − 20 °C in the laboratory.

### Stomach content analysis

At the laboratory, fish stomachs were thawed, the contents were rinsed with distilled water and were examined under a compound microscope. The food items were identified to the lowest possible taxonomic level. The identified diet items were counted and weighed to the nearest gram according to Hyslop [15]. The diet items were grouped into broader categories, namely brachyuran crab, mature penaeid shrimps, juvenile shrimps (post larvae and juvenile stages of all shrimp species), other shrimps (other mature shrimps), stomatopods, Teleostei (identified to family level) and digested materials. To determine the importance of each food item in the diet of each predatory fish species, the percent of prey-specific index of relative importance (%PSIRI) [36] was used. According to Brown et al. [36], %PSIRI is calculated using the following equation, %PSIRI = (%FO_i_ (%PN_i_ + %PW_i_))/0.5, where %FO_i_ is the percent frequency of occurrence (the number of stomachs containing prey *i* divided by the total number of stomachs with food); and %PN_i_ and %PW_i_ are the percent prey-specific abundances by number and weight respectively. The percent prey-specific abundances are the average percentage abundance of prey *i* by number (%PN_i_) or weight (%PW_i_). Overall interspecific variation in the diet of the fish species across the three estuarine zones, intraspecific variations of the diet at a spatial scale and in different size classes were assessed by main and pairwise permutational multivariate analysis of variance (PERMANOVA). In order to test whether the species exhibit ontogenetic changes in diet, the dietary data of successive 5 cm length classes of each species in each estuarine zone were used. The PERMANOVA tests were run based on the Bray–Curtis similarity matrices made from the square root transformed weight percentage dietary data. PRIMERv6 [37] with PERMANOVA + add-on module statistical packages [38] were used in these analyses.

### Stable isotope analysis

The dorsal white muscle tissues from 58 samples of *C. chrysophrys* and 60 samples of *E. malabaricus* collected in January and March 2015 were prepared for stable isotope analyses. The pieces of the dorsal muscle from individual fish were washed with distilled water and sun-dried. Thereafter, the samples were ground into fine-powder, packed into airtight plastic vials and kept in a desiccator until the time of analysis. Invertebrates, soon after being thawed, were sorted (under a dissection microscope) into various taxonomic groups and washed with distilled water. For large invertebrates, tissue samples were collected from the various body parts including the abdominal muscles of decapod shrimps, the claws of brachyura, the muscular foot of gastropods and the adductor muscle of bivalves. For amphipods and isopods individuals were pooled, sun-dried, ground into powder and packed for stable isotope analyses. For the sPOM, the top centimeter of the sediment was separated from the frozen core and prepared for isotopic analysis. The microphytobenthos samples and other autotroph samples were unfrozen and prepared for isotopic analyses. All samples prepared were then sent to the Iso-Analytical Laboratory in United Kingdom for stable isotope analysis.

In the stable isotope laboratory, the sample and references were weighted into tin capsules and loaded into an auto-sampler in sequence on a Europa Scientific elemental analyzer-isotope ratio mass spectrometry (EA-IRMS). Samples were combusted in an oxygen rich environment, raising the temperature in the region of the sample to ~ 1700 °C. The produced N_2_ and CO_2_ gases, after removing H_2_O, O_2_ and converting NO_x_ species to N_2_, were separated by packed column gas chromatography. The resulting separated gases were analyzed using the Europa Scientific 20-20 isotope ratio mass spectrometer (Sercon Ltd. UK). The samples for microphytobenthos, sPOM, periphyton and invertebrates were acid washed with 1 M hydrochloric acid to remove inorganic carbonate prior to carbon stable isotope analysis. Then, the samples were neutralized by repeatedly washing with distilled water and subsequently oven dried at 60 °C. During analysis, samples were analyzed in batches being interspersed with working references (soy protein, tuna protein), calibrated with the International Atomic Energy Agency (IAEA) standards (IAEA-C7, IAEA-CH-6 and IAEA-N-1). The results of isotopic values were expressed in standard δ notation, as part per thousand (‰) relative to PeeDee Belemnite carbon for δ^13^C and atmospheric N_2_ for nitrogen (δ^15^N) according to the following equation:

$$\delta {\text{X}} = \left[ {\left( {\frac{{{R_{sample}}}}{{{R_{standard}}}}} \right) - 1} \right] \times \,{10^3}$$

where X = ^15^N or ^13^C, R = the ratio ^13^C/^12^C or ^15^N/^14^N [15].

The isotopic results of the fish samples were not normalized for lipid correction as their C:N ratios were below 3.5, an indication of lipid levels that do not adversely skew the isotopic results. For the tissue samples of invertebrates, the observed C:N ratios were above 3.5, and thus their isotopic results were lipid corrected using the lipid normalization models [39]. The following mathematical lipid normalization model was applied as described by Post et al. [39].

$${\delta^{13}}{{\text{C}}_{\text{normalized}}} = {\delta^{13}}{{\text{C}}_{\text{untreated}}} - 3.32 + 0.99 \times {\text{C}}{:}{\text{N}}$$

where δ^13^C_untreated_ are the δ^13^C values that were directly measured in species, and C:N ratio is calculated from direct measurement of C and N during stable isotope analysis. Differences in δ^13^C and δ^15^N signatures between and within the fish species at spatial scale were analyzed using one-way ANOVA. Linear regression was applied in order to determine if the δ^15^N and δ^13^C values were changing with increasing fish length. Prior to the implementation of ANOVA and linear regression, data were checked for normality. No statistical test was carried out to find the differences in δ^15^N and δ^13^C ratios within and among primary producers and potential prey due to small number of samples; they were only collected to trace their isotopic signatures in the nutrition of the fish.

The relative proportion contributions of all autotrophs to the nutrition of fish in each estuarine zone were estimated using the isotope mixing model Stable Isotope Analysis in R package (SIAR) version 3.3.0 [40]. More than one category of potential autotrophs with similar isotopic composition were combined as the model does not fit well when the isotopic values of food sources do not differ [41]. The trophic position of consumer is important when running the model [41], thus the estimated trophic levels (TL) of about 3.0 for the *C. chrysophrys* and 3.2 for the *E. malabaricus* were used. These TLs were estimated following the method of Post [42] using *Uca* species, the primary consumer invertebrate as the baseline. *Uca* crabs (family Ocypodidae) were selected in this case because they were abundantly caught in all three sampling zones and largely feed on benthic algae and diatoms [43, 44]. The formula used was TLi = 2 + (δ^15^N_i  − _δ^15^N_Tbase_/3.0) where: TLi = trophic level of fish species i, δ^15^N_i_ = is the mean nitrogen isotopic value for fish species i, δ^15^N_Tbase_ = mean nitrogen isotopic value of *Uca* species that was considered as trophic level 2 in each sampling zone. The trophic fractionation factors (TFF) of consumers used were 0.5 for δ^13^C and 3.0 for δ^15^N following the recommendation of Abrantes et al. [5], McCutchan et al. [45] and Vanderklift and Ponsard [46].

The Bayesian mixing models were also used to quantify the contributions of various preys to the diet of the predatory fish species. The stomach content analyses and available literature were used to identify the potential prey food sources that were preyed upon. The models were run separately for each estuarine zone by using the isotopic values of the predatory fish and their potential prey collected in the three respective zones. Also, before running the model, the δ^13^C and δ^15^N of the predatory fish were corrected for trophic fractionations using the same TFF values as applied above. The outputs of the model for each category of autotrophic and prey contributions to each fish species were presented as mode with 95% credibility interval for variance as recommended by Parnell et al. [47].

The Layman metrics mainly used to describe the trophic structure and trophic niches [47, 48] were calculated using Stable Isotope Bayesian Ellipses in R (SIBER) packages version 4.4 [40]. These metrics among others were the range of δ^13^C values (CR) for measuring trophic diversity of the basal resources, standard ellipse area (SEA) and total area (TA). The TA represented by convex hulls is used to estimate the isotopic niche width of each fish species and their degree of resources segregation in sampling zones. The calculated standard ellipse areas in δ^13^C–δ^15^N bi-plot space, corrected for small sample size (SEAc) (n > 30) represented the bivariate standard deviation of the data and encompassed about 40% of the data [44]. Convex hull area (TA) included all samples of each species in the δ^13^C–δ^15^N space, represents the total niche space occupied by the group. For statistical testing of the isotopic niche width of fish species, Bayesian standard ellipse area (SEAb) were estimated [48]. Both SEAc and the estimated SEAb (after fitting Bayesian multivariate normal distributions to each species in the dataset) measure the feeding niches and indicate the degree of niche overlap among the species. Both SEAc, SEAb are unbiased with respect to sample size and display more uncertainty with smaller sample size [48, 49].

## Results

### Fish diet

A total of 113 stomachs of *C. chrysophrys* and 107 of *E. malabaricus* were found with food in all three estuarine zones. Twenty-two stomachs of *C. chrysophrys* and 57 of *E. malabaricus* were empty (Table 1). Total length of *C. chrysophrys* ranged from 12 to 38 cm, whereas that of *E. malabaricus* ranged from 10 to 54 cm. In all estuarine zones, the percent frequency of occurrence (%FO = 60.85) and prey-specific index of relative importance indicated that juvenile shrimps dominated the diets of *C. chrysophrys* (%PSIRI = 51.37%) (Table 1). Teleostei, mainly clupeids and engraulid species, were also the most highly consumed food items by *C. chrysophrys*. In contrast, the diet of *E. malabaricus* was dominated by brachyura (%PSIRI = 38.80%), followed by juvenile shrimps (%PSIRI = 25.57%) and Teleostei (%PSIRI = 23.29%) (Table 1). The most frequently consumed prey by *E. malabaricus* among others included, the members of Clupeidae and Mugilidae families (Table 1).

**Table 1:** Diet composition of *Carangoides chrysophrys* and *Epinephelus malabaricus* from the Pangani Estuary

Food items	*C. chrysophrys*				*E. malabaricus*			
	%FO	%PN_i_	%PW_i_	%PSIRI	%FO	%PN_i_	%PW_i_	%PSIRI
Brachyura	12.5	54.70	74.12	8.05	50.00	77.44	77.75	38.80
Juvenile shrimps	60.85	91.18	78.42	51.37	31.13	86.30	77.98	25.57
Teleostei–Clupeidae	14.42	80.96	91.08	12.41	17.92	69.62	75.74	13.03
Teleostei–Mugilidae	3.85	53.47	78.45	2.54	10.38	65.76	77.59	7.44
Teleostei–Engraulidae	15.38	63.67	73.66	10.56	1.89	50.00	24.56	0.70
Teleostei–Gobiidae	0	0	0	0	3.77	56.25	55.99	2.12
Unidentified fish	0.96	100.00	100.00	0.96	0	0	0	0
Penaeid shrimps	14.42	79.85	89.15	12.19	6.60	69.52	79.39	4.92
Other shrimps	1.97	100.00	100.00	1.92	3.77	70.83	70.50	2.67
Stomatopoda	0	0	0	0	6.60	72.38	71.87	4.76
Total				100.00				100.00
Total empty stomachs (%)	22 (16.30)				57 (34.76)			
Total stomachs with food (%)	113 (83.70)				107 (65.23)			

*%FO* percentage frequency of occurrence, *%PN*_*i*_ percent prey-specific abundance, *%PW*_*i*_ percent prey-specific weight of food item in the stomachs of each species and *%PSIRI* percentage of prey-specific index of relative importance of each food item in the diet of the predatory fish species

Most food items utilized by both fish species along the longitudinal salinity gradient were more or less similar but they differed in the levels of importance as indicated by %PSIRI. Juvenile shrimps were the most important diet consumed by *C. chrysophrys* across the estuarine zone (Table 2). The %PSIRI values for the Teleostei showed that engraulids were more or less caught equally by *C. chrysophrys* throughout the estuarine zones whereas, clupeids were frequently caught in the upper and middle zones (Table 2). Regarding the trend of food items caught by *E. malabaricus* across the estuarine zones, brachyura were frequently ingested in the middle (%PISIRI = 40.62) and lower (%PISIRI = 41.78) estuarine zones when compared with the upper estuarine zone (%PSIRI = 34.81) (Table 2).

**Table 2:** Prey-specific index of relative importance (%PSIRI) for the predatory fish in the Pangani estuarine zones

Food items	*C. chrysophrys*			*E. malabaricus*		
	Upper zone	Middle zone	Lower zone	Upper zone	Middle zone	Lower zone
Brachyura	6.14	7.65	10.37	34.81	40.62	41.78
Juvenile shrimps	50.59	49.23	54.90	29.56	21.35	25.69
Teleostei–Clupeidae	14.78	17.78	3.13	10.45	17.34	11.36
Teleostei–Mugilidae	6.67	0.70	1.08	10.80	10.24	1.19
Teleostei–Engraulidae	10.38	8.09	13.98	1.10	0	0.29
Teleostei–Gobiidae	0	0	0	1.32	1.34	3.71
Unidentified fish	3.33	0	0	0	0	0
Penaeid shrimps	4.78	14.16	16.54	2.78	4.19	7.85
Other shrimps	3.33	2.38	0	3.63	0	4.35
Stomatopoda	0	0	0	5.56	4.93	3.78
Total	100.00	100.00	100.00	100.00	100.00	100.00

The two ways PERMANOVA revealed the high level of variation in the diets between *C. chrysophrys* and *E. malabaricus* across the three estuarine zones (*p* = 0.001, pseudo-*F* = 15.81) but it did not show any interaction between the species and sampling zones (*p *> 0.05; pseudo-*F* = 0.43). Lack of interaction for the fish species and estuarine zones factors, was further shown by the pair-wise PERMANOVA tests which indicated no spatial intra-specific diet variation of both species for each pair of zonal comparison (*p *> 0.05; t ≤ 1.07). With respect to fish size, one-way PERMANOVA confirmed that the two species did not show a significant shift in diet as they grew throughout the estuarine zones (*p *> 0.05).

### Stable isotopes of autotrophic sources

The carbon isotope values (δ^13^C) for the autotrophic resources were variable (Table 3). The mean δ^13^C of sPOM slightly increased from the upper estuarine reaches towards the estuary mouth (Table 3). Its nitrogen isotope (δ^15^N) values were relatively similar in all three zones. The macro-algae *Sargassum* species and seagrasses were only abundant near the estuary mouth and comprised the more enriched δ^13^C ratio when compared with the δ^13^C ratio of microphytobethos, periphyton and green filamentous algae. Thus, the δ^13^C values for all autotrophic sources in all sampling zones ranged from the most enriched sources—sea grass, macro-algae (*Sargassum* species) and C_4_ plant, intermediate sources—periphyton, microphytobenthos, green filamentous algae and sPOM to the most depleted sources—C_3_ vascular plants. Overall, microphytobenthos, macro-algae (*Sargassum* species) and sea grasses were the autotrophic sources that contained the lowest δ^15^N values in all estuarine zones (Table 3).

**Table 3:** Stable isotopes of autotrophic sources (mean  ‰ ± standard deviation) in Pangani estuary

Primary food source	Upper estuarine zone		Middle estuarine zone		Lower estuarine zone	
	δ^13^C	δ^15^N	δ^13^C	δ^15^N	δ^13^C	δ^15^N
Sediment POM	− 23.9 ± 0.7 (3)	6.8 ± 0.2 (3)	− 23.3 ± 0.5 (3)	6.9 ± 0.2 (3)	− 22.9 ± 0.7 (3)	6.4 ± 0.3 (3)
Periphyton	− 23.4 ± 0.3 (3)	5.4 ± 0.4 (3)	− 22.75 (2)	6.2 (2)	− 19.9 (2)	6.9 (2)
Microphytobenthos	− 20.6 (1)	3.7 (1)	− 20.5 (1)	3.6 (1)	− 20.2 (1)	4.0 (1)
Filamentous green algae	–	–	− 22.0 (2)	6.0 (2)	− 21.6 ± 1.4 (4)	6.1 ± 0.3 (4)
Macro-algae (*Sargassum* sp.)	–	–	–	–	− 16.8 (2)	3.0 (2)
Sea grass	–	–	–	–	− 13.7 ± 1.0 (3)	4.0 ± 0.2 (3)
C_4_ grasses	− 12.9 ± 0.3 (5)	5.2 ± 1.2 (5)	–	–	–	–
C_3_ Mangrove plant	–	–	− 28.4 ± 1.2 (8)	5.4 ± 1.8 (8)	− 28.7 ± 0.8 (7)	3.9 ± 1.9 (7)
C_3_ plant	− 28.1 ± 1.3 (5)	6.5 ± 2.5 (5)	− 27.9 (1)	5.71 (1)	–	–

Sample size = n for each autotrophic group is indicated in brackets. When n < 3 standard deviation values are not given

*δ*^*13*^*C* Carbon isotope ratio, *δ*^*15*^*N* nitrogen stable isotope ratio, *sPOM* surface sediment particulate organic matter

### Stable isotopes of fish and potential prey

With the exception of mugilid and clupeid prey fish, the results revealed a slight variation in δ^13^C among the potential prey in all estuarine zones (Table 4). In the upper estuarine zone, the mean δ^13^C value of gastropods was similar to that of isopods, brachyura and shrimps. In the middle portion of the estuary, the δ^13^C values of gastropods, bivalves, amphipods and brachyura were also similar. In the lower portion of the estuary, similar δ^13^C values among the groups of potential prey included gastropods, bivalves, isopods and amphipods (Table 4). Clupeid fish, as potential prey for the predatory fish species, presented enriched δ^13^C values compared to all examined prey categories, and were more or less similar across the three estuarine zones. In contrast, mugilid prey fish showed the most depleted δ^13^C values compared to all prey collected from the upper, middle and lower portions of the estuary. Furthermore, mugilids showed the most elevated δ^15^N ratio among all prey items in all estuarine zones (range 13.0 to 12.4‰). The δ^15^N values of clupeids, shrimps and brachyura were very close to each other in each sampling zone and ranged from 9.8‰ to 11.1‰. On the other hand, the δ^15^N values of gastropods were the lowest among all invertebrates (Table 4).

**Table 4:** Stable isotopes of invertebrates and fish studied (mean  ‰ ± standard deviation) in the Pangani estuary

Taxa	Upper estuarine zone		Middle estuarine zone		Lower estuarine zone	
	δ^13^C	δ^15^N	δ^13^C	δ^15^N	δ^13^C	δ^15^N
Bivalves	–	–	− 18.1 ± 1.2 (5)	8.9 ± 0.5 (5)	− 16.8 ± 0.3 (4)	8.6 ± 0.5 (4)
Gastropods	− 20.1 ± 2.3 (6)	7.0 ± 1.1 (6)	− 18.2 ± 2.4 (3)	6.7 ± 0.3(3)	− 17.3 (2)	6.4 (2)
Amphipods	–	–	− 18.9 (2)	9.0 (2)	− 18.1 (2)	10.0 (2)
Isopods	− 20.9 (1)	8.5 (1)	− 19.6 (1)	8.4 (1)	− 18.4 (1)	7.8 (1)
Brachyura	− 19.6 ± 1.9 (7)	10.2 ± 0.5 (7)	− 18.6 ± 1.5 (9)	9.8 ± 0.8 (9)	− 18.5 ± 1.7 (8)	9.8 ± 0.6 (8)
Penaid and palaemonid shrimps	− 20.2 ± 1.1 (6)	10.5 ± 0.7 (6)	− 20.7 ± 0.8 (4)	10.6 ± 0.4 (4)	− 18.9 ± 0.5 (5)	10.4 ± 0.6 (5)
Clupeids	− 17.8 ± 0.6 (19)	10.8 ± 0.4 (19)	− 17.8 ± 0.5 (20)	11.1 ± 0.7 (20)	− 17.6 ± 0.5 (20)	10.9 ± 0.5 (20)
Mugilids	− 23.2 ± 0.7 (20)	13.0 ± 0.5 (20)	− 22.8 ± 1.0 (22)	12.8 ± 0.7 (22)	− 22.3 ± 0.8 (22)	12.4 ± 0.8 (22)
*C. chrysophrys*	− 19.7 ± 0.9 (19)^a,^*	13.5 ± 0.5 (19)^a,^*	− 19.3 ± 1.1 (20)^ab,^*	13.5 ± 0.5 (20)^a,^*	− 18.9 ± 1.1 (19)^b,^*	13.3 ± 0.5 (19)^a^,*
*E. malabaricus*	− 19.1 ± 1.0 (21)^a,^*	14.0 ± 0.9 (21)^a,^**	− 18.8 ± 0.8 (19)^a,^*	13.9 ± 0.6 (19)^a,^**	− 17.5 ± 0.6 (20)^b,^**	13.9 ± 0.4 (20)^a,^**

*δ*^*13*^*C* Carbon isotope ratio, *δ*^*15*^*N* nitrogen stable isotope ratio

^a^ and ^b^ indicate significant differences revealed by ANOVA for each species across the sampling zones; * and ** indicate interspecific significant differences revealed by ANOVA. Sample size = n are given in brackets. When n < 3 standard deviation values are not given

The ANOVA results revealed that the δ^13^C values of *C. chrysophrys* and *E. malabaricus* were significantly different in the middle and lower portions of the estuary but not in the upper estuarine zone (Table 4). The δ^15^N of both species were statistically different in all three estuarine zones (Table 4). Across the estuarine zones, each fish species displayed insignificant changes in δ^15^N ratio (ANOVA, *p* > 0.05). The δ^13^C values of *C. chrysophrys* were slightly different between samples caught from the upper and lower estuarine reaches (ANOVA, F = 5.86, *p* < 0.05) but similar in δ^13^C ratio of samples caught in the other two possible zonal comparisons. The δ^13^C ratio of *E. malabaricus* slightly increased with increasing distance from the upper estuarine reaches towards the estuary mouth (Table 4). The δ^13^C values of this species were different between the upper and lower zone (ANOVA, F = 43.37, *p* < 0.001), as well as the middle and lower part of the estuary (ANOVA, F = 33.7, *p* < 0.001). For the pooled estuarine zonal δ^13^C and δ^15^N data, the two predatory fish species were isotopically distinct (ANOVA, F ≤ 19.9, *p* = 0.0001).

The linear regression results for the pooled isotopic data across the estuarine zones, showed no significant relationship between fish length and δ^13^C or δ^15^N ratios of *C. chrysophrys* (*F*_1, 53_ ≤ 0.92, r^2^ ≤ 0.02, *p* > 0.05). For *E. malabaricus*, the linear regression of the combined estuarine zonal data revealed no significant relationship between fish size and δ^15^N (*F*_1, 58_ = 1.3, r^2^ = 0.02, *p* > 0.05). However, *E. malabaricus* exhibited a weak positive linear relationship between length and δ^13^C values (F_1, 58_ = 5.0, r^2^ = 0.08, *p* = 0.03). Regarding the linear regression results of each species in every estuarine zone, it was found that both species had no relationship of fish size and δ^13^C or δ^15^N values (F ≤ 4.2, r^2^ ≤ 0.2, *p* > 0.05).

### Autotrophic sources supporting the nutrition of fish

**Fig. 2:**
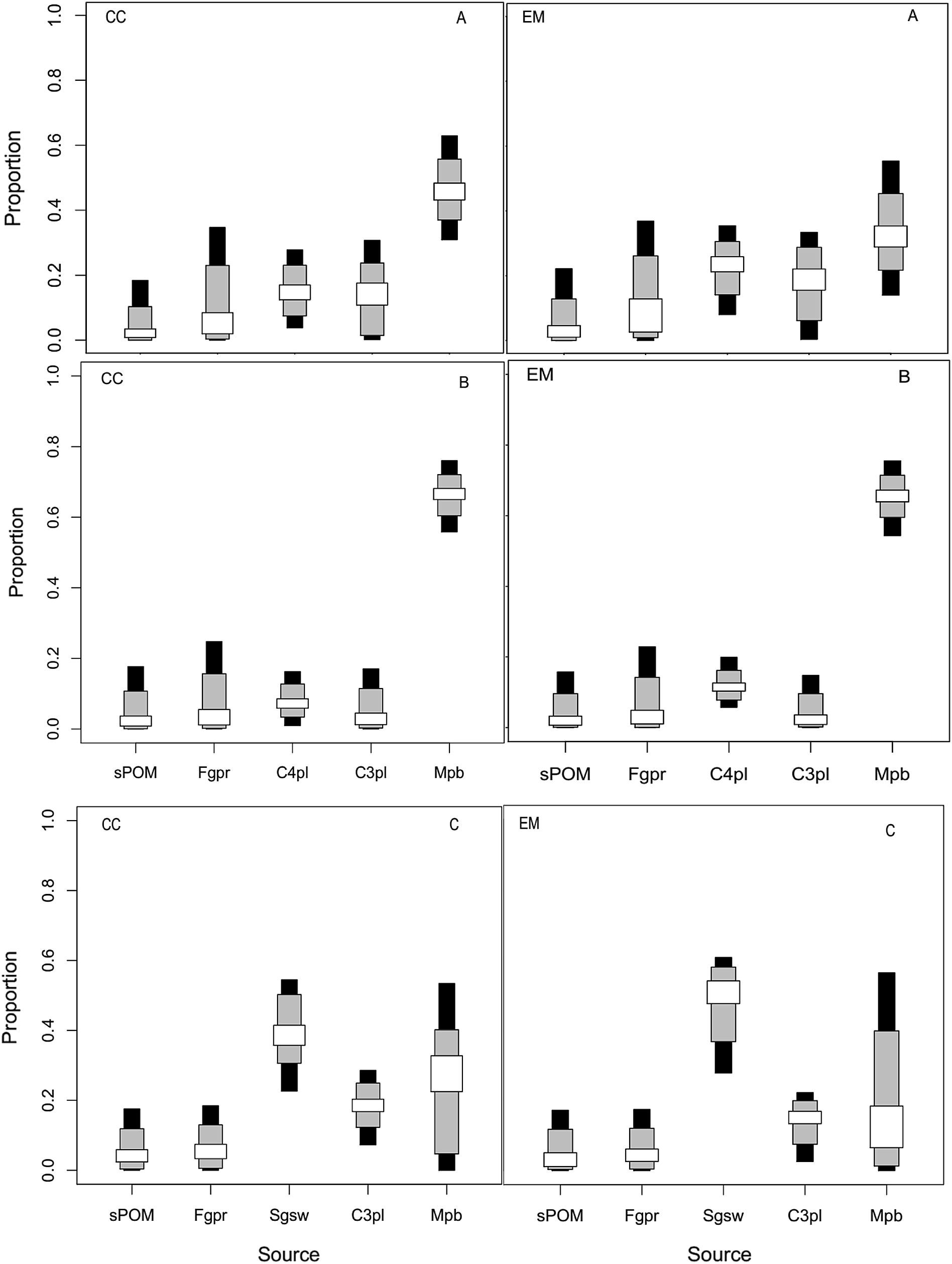
Relative proportion contribution of autotrophic sources to the nutrition of fish in the Pangani Estuary. The box plots represent 25% (white), 75% (grey) and 95% (black) credibility intervals of autotrophic sources to the fish nutrition, CC, *Carangoides chrysophrys* and EM, *Epinephelus malabaricus* caught in **a** upper, **b** middle and **c** lower estuarine zones. Autotrophic sources included *sPOM* surface particulate organic matter (sPOM), *Fgpr* combined filamentous green algae and periphyton, *Sgsw* combined macro-algae (*Sargassum* species) and sea grass, *C4pl* C_4_ plants, *C3pl* C_3_ plants including mangroves, and *Mpb* microphytobenthos

The findings revealed that *C. chrysophrys* and *E. malabaricus* differed in the proportions of primary producers they rely on in the Pangani estuary. In the upper estuarine reaches, microphytobenthos was the dominant source of energy both *C. chrysophrys* and *E. malabaricus* relied on (Fig. 2). The mode estimates of the relative contribution of microphytobenthos at 95% credibility interval (CI) were 45.3% (30–64% CI) for *C. chrysophrys* and 31.2% (15–56% CI) for *E. malabaricus*. The C_4_ grasses were the second most important contributors to the nutrients and energy required by *C. chrysophrys* (14.5% mode, 4–28% CI) and *E. malabaricus* (24.8%, 8–35% CI). The C_3_ plant producers were also as important as C_4_ grass plants for both species; its mode contribution ranged from 17.3 to 18.7% (0.1–33% CI). sPOM and periphyton showed CI that started at zero, and therefore can be considered unimportant for the overall nutrition of predatory fish (Fig. 2).

In the middle part of the estuary, microphytobenthos were again the main autotrophic sources, and their percent contributions were slightly higher [*C. chrysophrys* (56–76% CI), *E. malabaricus* (54–75% CI)] when compared to those from the upper part of the estuary (Fig. 2). The second most important food source assimilated by both species were C_4_ grasses but the percent contribution were slightly lower than that revealed in the upper estuarine zone (Fig. 2). sPOM, periphyton, filamentous green algae and C_3_ plants were less important (Fig. 2). In the estuarine mouth zone, seagrasses and macro-algae (*Sargassum* species) were the key food sources for *C. chrysophrys* and *E. malabaricus* (21–55% CI and 29–60% CI, respectively). The next important food source were C_3_ plants for both *C. chrysophrys* and *E. malabaricus* (Fig. 2). Microphytobenthos indicated the broader percent contribution to the nutrition of both fish species in the lower portion of the estuary. However their importance were slightly lower when compared with their position in the other sampling zones, as their credible contribution lay between 0% to about 55% (Fig. 2).

### Important prey supporting the nutrition of fish

In all three estuarine zones, clupeids and mugilid species were highly assimilated by *C. chrysophrys* followed by brachyura and shrimps in the estuary (Table 5). Mugilid prey fish presented the most elevated proportional contribution of about 27.6% mode to the overall nutrition of *C. chrysophrys* in the lower estuarine zone compared to that of the other zones, where the clupeid prey percent contributions were higher (Table 5). On the other hand, clupeids were the most utilized prey by *E. malabaricus* throughout the Pangani estuarine system (Table 5). Mugilids were the second most important prey for the nutrition of *E. malabaricus* though, their mode percent contributions were decreasing from the upper (18.5%) towards the estuary mouth zone (7.4%) (Table 5).

**Table 5:** Proportional contributions of food items for *C. chrysophrys* and *E. malabaricus* in Pangani estuary

Potential prey	*C. chrysophrys*					
	Upper estuarine zone		Middle estuarine zone		Lower estuarine zone	
	Mode	CI	Mode	CI	Mode	CI
Gastropoda	4.45	0–19	1.49	0–16	2.44	0–18
Isopoda	3.21	0–24	1.91	0–21	9.49	0–25
Brachyura	21.00	0–39	22.79	0–43	18.18	0–38
Caridea and penaeidae shrimps	19.81	0–39	18.70	0–41	20.78	0–36
Clupeidae	22.78	6–36	27.41	11–27	19.94	0–31
Mugilidae	20.44	10–30	14.16	5–26	27.56	15–38

Potential prey	*E. malabaricus*					
	Upper estuarine zone		Middle estuarine zone		Lower estuarine zone	
	Mode	CI	Mode	CI	Mode	CI
Gastropoda	1.03	0–11	1.00	0–9	0.74	0–5
Isopoda	1.17	0–14	1.04	0–11	0.68	0–6
Brachyura	17.27	0–40	3.28	0–35	1.33	0–17
Caridea and penaeidae shrimps	10.04	0–37	2.93	0–33	1.88	0–22
Clupeidae	36.66	0.1–55	47.89	30–64	74.30	6–86
Mugilidae	18.51	0.1–29	16.57	6–25	7.40	2–14

Relative contributions of dietary sources to predatory fish reported as mode (central tendency) with 95% CI = Bayesian credible interval

### Trophic niche characteristics of fish

**Fig. 3:**
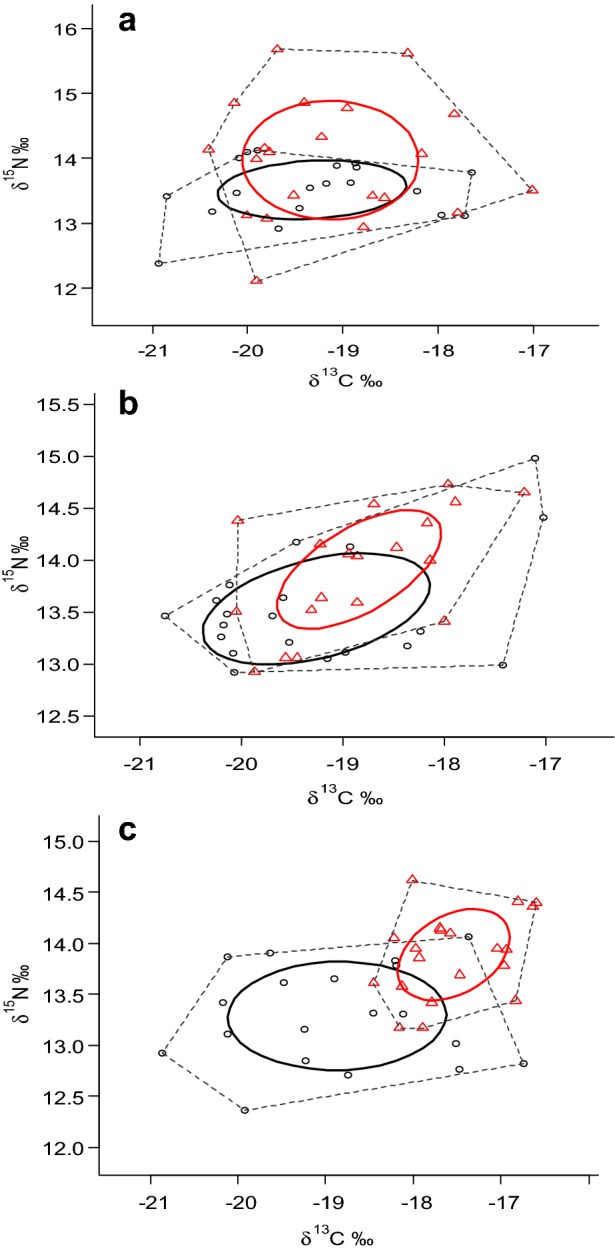
Standard ellipse areas (‰) and convex hulls of the predatory fish species in the Pangani estuarine zones. Standard ellipse areas (‰) are corrected for small sample sizes denoted as SEAc in solid line cycles that comprised about 40% of isotopic data, presented as ○ (open points) for *Carangoides chrysophrys* and ∆ open (triangle points) for *Epinephelus malabaricus*. Convex hull areas (TA) presented as dotted lines indicate the total niche space occupied by each species, The letters, **a**, **b** and **c** indicate upper, middle and lower estuarine zones respectively

The δ^13^C range of *C. chrysophrys* was decreasing from the lower zone (4.13‰) towards the upper, with the lowest salinity, zone (3.3‰) (Table 6). The opposite was noticed for the δ^13^C range of *E. malabaricus* that was higher in the upper estuarine zone (3.4‰) and lower in the lower, with high salinity, zone (1.9‰). The Bayesian estimated standard ellipse area (SEAb) of the *C. chrysophrys* was significantly higher in the lower estuarine zone (2.1 ± 0.5‰) compared to the middle (1.6 ± 0.6‰) and upper zone (1.4 ± 0.4‰^2^, ANOVA F = 9.39, *p* < 0.05; Table 6; Fig. 3). However, standard ellipse area (SEAc) and thus the trophic niches of this species between the upper and middle zone as well as between the middle and lower sampling zone were similar (ANOVA, *p* > 0.05). This was different for the *E. malabaricus* since SEAb was significantly increasing from the river mouth towards the upper part of the estuary (ANOVA F > 22, *p* < 0.05; Table 6; Fig. 3). The trophic niche of *E. malabaricus* was statistically higher in the upper part of the estuary (F = 24.76, *p *= 0.001) and lower in both the middle and the zone near the estuary mouth (ANOVA, F ≥ 4.8, *p* ≤ 0.05) compared to the corresponding trophic niches of *C. chrysophrys* (Table 6). Despite the fact that both species demonstrate overlapping SEAc and convex hulls in all three estuarine zones, these overlapping spaces were larger in the upper and middle portions, and relatively small in the lower portion of the estuary (Fig. 3).

**Table 6:** Isotopic niche data of *Carangoides chrysophrys* and *Epinephelus malabaricus* in the Pangani estuarine zones

Isotopic niche data	Upper estuarine zone		Middle estuarine zone		Lower estuarine zone	
	*C. chrysophrys*	*E. malabaricus*	*C. chrysophrys*	*E. malabaricus*	*C. chrysophrys*	*E. malabaricus*
CR (‰)	3.28	3.40	3.73	2.84	4.13	1.85
SEAc (‰^2^)	1.45	2.74	1.77	1.20	1.84	0.74
SEAb (mean ‰^2^ ± SD)	1.35 ± 0.37	2.51 ± 0.32	1.64 ± 0.56	1.13 ± 0.17	2.06 ± 0.50	0.72 ± 0.19
TA	3.67	7.59	4.62	3.35	4.88	1.93

CR = the δ^13^C range (difference between the smallest and largest values of δ^13^C), SEAc = the standard ellipse area (corrected for small sample sizes), and SEAb = the estimated mean Bayesian standard ellipse area

## Discussion

The isotopic mixing models and dietary results revealed that the high-speed swimming and roving *C. chrysophrys* and the less mobile *E. malabaricus* consumed similar types of prey but in different proportions, in the various reaches of the Pangani estuarine system. The isotopic mixing models also verified that the two species differ in the isotopic niche width they occupy, across the longitudinal estuary gradient. Differences in the amount of prey consumed are likely to be justified by prey distribution in the feeding habitats (i.e., benthic and benthopelagic), prey catchability, feeding strategies and energy requirements of each species [12, 50]. Because of the high swimming speed laterally and longitudinally, *C. chrysophrys* is capable of capturing highly mobile prey that are abundant all over the water column, whereas, the sedentary nature and ambush foraging strategy of the benthic *E. malabaricus* [7, 26, 31, 51] enables it to capture large amounts of less mobile zoobenthos.

The differences in foraging habitats (defined by depth) and foraging strategy of these two predatory fish species, typifies the lower δ^13^C values of *C. chrysophrys* when compared to that of *E. malabaricus* in all three estuarine zones. It is known that the mid-water and pelagic food sources tend to display more negative δ^13^C ratios whereas, the benthic food sources exhibit less negative δ^13^C ratio [52]. Thus, the interspecific variation in δ^13^C values probably resulted from the fact that *C. chrysophrys* depends on the mid-water and pelagic prey in addition to benthic, which is reflected in the more negative δ^13^C values compared to the mostly benthic (and less negative δ^13^C) signal of *E. malabaricus*. It was, however, noted that the most important preys consumed by the two species as revealed by the dietary indices, were to some extent different from the most assimilated prey by the same species, as shown by isotopic mixing models. The overemphasized prey category of brachyura and shrimps as indicated by stomach content analysis over small bony fish revealed by isotopic mixing models suggests that either the two species rely on prey types whose abundance vary greatly, or these prey types share similar basal nutritional sources that were not affected by season. Such justifications are supported by the long-term, isotopic signal dietary tracers that reveal diets consumed by the species for the past 3 months. More so, the high occurrence of large sized preys like brachyura in the diet of *E. malabaricus* in contrast to numerous juvenile shrimps predated by *C. chrysophrys* could be correlated to differences in their mouth morphology [30, 53].

The varied δ^13^C values of *E. malabaricus* across the estuarine zones further demonstrates that the feeding of this species is site-specific and localized due to a limited foraging movement among the estuarine zones [54, 55]. Moreover, the differences in the prey foraging strategies of *C. chrysophrys* and *E. malabaricus* may have contributed to the interspecific differences in δ^13^C and δ^15^N signatures, indicating trophic niche differentiation and co-existence in the estuary. The difference in the percent contributions of mugilids and clupeids to the nutrition of *C. chrysophrys* and *E. malabaricus* suggests that these species differ in the trophic pathways of acquiring their carbon sources and nutrients. This is because mugilids form the component of the benthic food web; and clupeid the component of the planktonic food web. Provided that these predatory fish species showed different proportions of relying on mugilid and clupeid diet, they exploit differently nutrients and energy channels from pelagic and benthic food webs. This situation reduces fragility and increases trophic connectivity of estuarine food web structure [9, 10].

Furthermore, the findings suggest that there could be a facultative mutualistic feeding interaction between *C. chrysophrys* and *E. malabaricus*. This means that the high speed, patrolling technique of seizing preys on the bottom of the sediment by *C. chrysophrys* tends to disturb the epibenthic organisms like gobies, shrimps and brachyura. Such disturbance predisposes these prey items to be readily caught by *E. malabaricus*, a predator that seizes its prey through sit-and-wait ambush strategy. Apart from that, the low salinity portion of the estuary which is more turbid and structurally complex, probably enhances the prey-capture success by *E. malabaricus*. The structurally complex habitat provide enhanced cover that reduces the possibilities of predators to be detected by the preys [55]. This situation is perhaps connected with the observed broader isotopic niche space of *E. malabaricus* when compared to that of *C. chrysophrys* in the upper estuarine zone. The catchability of prey by *C. chrysophrys* is likely to be affected by the turbid conditions which lowers its visibility [56] in the upper estuarine zone.

The observed changes in the main autotrophic sources *C. chrysophrys* and *E. malabaricus* relied on in the three estuarine zones, could be underpinned by the selectivity of the trophic basal sources by organisms that are preyed on by predatory fish. Selectivity of the autotrophic resources by primary consumers is largely driven by digestibility of the resources. According to Mann [57] and Dias et al. [58] terrestrial C_3_ and C_4_ plant food sources are refractory, less labile relative to micro- and macro-algae food sources. The reliance on sea grass and macro-algae (*Sargassum* species) by *C. chrysophrys* and *E. malabaricus* in the lower sampling zone, indicates that their preys most probably feed on and assimilate the autotrophic source that is readily abundant. Matich et al. [6] also observed that *Carangoide*s species and other coastal predatory species in northern Tanzania reflected the isotopic signals of the mixed algae and sea grasses due to the high abundance of these autotrophic sources.

Notably, the Panagni estuary is shallow [19] with wide intertidal mudflat that supports microphytobenthic production [59, 60] while marine microalgae are washed into the estuary by strong tidal flows. This is probably reinforced by the reduced freshwater flow due to water use for three hydro-electric power generation, and water abstraction for irrigation schemes upstream the Pangani River catchment [19, 20]. On the other hand, the δ^13^C values representing C_4_ plant as the second most assimilated autotrophs by fish species in the upper and middle zones, are likely to be a combination of the isotopic signals derived from both C_4_ plant and sea grasses from the lower part of the estuary. This is due to the fact that the δ^13^C values of these two autotrophs are relatively similar. This has an implication to the isotopic signals of the non-stationary *C. chrysophrys* and preys such as clupeids, engraulids and shrimps that frequently and unlimitedly move across the estuarine zones. This situation can also confirm the limitation of using isotopic values for the study of the dietary sources of estuarine consumers at fine spatial scales.

Unlike *E. malabaricus,* the decreasing trends of niche breadth (measured by carbon isotopic range) and isotopic niches of *C. chrysophrys* from the lower zone towards the upper estuarine zone, further highlight that, the two species differ in salinity and turbidity tolerances accompanying their feeding habitats. These trends suggest that *C. chrysophrys* migrates to the middle and upper estuarine zones to avoid high risk of predation and other anthropogenic disturbances that is common along the coast and township of Pangani. On the other hand, the larger isotopic overlapping space in the middle zone relative to that of the upper and lower estuarine zone is probably due to potential inter-specific competition. Because of potential inter-specific competition in the middle zone, the fish species can easily move out of that zone to either the upper or lower zones to reduce competition. This situation is probably linked with the broader isotopic niche of *E. malabaricus* in the upper estuarine reaches, which is the result of the species escaping potential competition in the middle zone and disturbances in the lower estuarine zone.

The dominance of benthic estuarine and marine microalgae in the nutrition of the fish species is probably an indication that there are lower connectivity options between the estuarine nursery food webs and the terrestrial or freshwater food webs. In this case, fragmentation of the food web is likely to occur, a situation which reduces the ecosystem complexity while weakening the preliminary nursery function of the estuaries. The situation is intensified by increasing anthropogenic fresh water use upstream of the estuary, as well as disturbances related to the local fishermen that frequently use the estuary as a fishing ground. Thus, the responsible authorities of the Pangani River Basin are advised to manage, and thus reduce all anthropogenic activities that are currently threatening the estuarine ecosystem.

The present study also demonstrates that not all estuarine benthic predatory fish communities and their prey, reflect the estuary gradient difference in δ^13^C signals of primary food sources [61]. Instead, this depends on the distance in between the sampling points and the foraging movement pattern of the animals. As for the case of δ^13^C ratio of the high mobile *C. chrysophrys,* our findings concurred with the study by Selleslagh et al. [62] who revealed that within the fine scale estuarine sampling points, no significant isotopic differences can be detected. Moreover, only site fidelity juvenile predatory fish species such as *E. malabaricus* [55] are able to show the isotopic signals similar to that of the basal nutritional sources found in the habitats the species is caught [62]. Likewise, our isotopic results displayed similar patterns reported by Claudino et al. [63] who emphasized that some fish and decapod invertebrates incorporate the most abundant autotrophic sources in habitats they occur. Claudino et al. [63] further showed that consumers near the estuary mouth, acquire their nutrients derived from macro-algae and seagrass, in contrast to the consumers from the upper estuarine zone that accumulate their nutrients from mangrove and macro-algae.

## Conclusions

The study described how the two juvenile predatory species, *C. chrysophrys* and *E. malabaricus* were spatially and trophically connected to each other, despite differences in their positions when feeding in the water column of the Pangani estuarine system. It was revealed that both species consume small fish, shrimps and brachyura constituting similar and narrow trophic resources base. The differences in their diets relied on the proportions of each prey consumed by individual species. The species differed significantly in their isotopic trophic niches but with considerable dietary overlap. The spatial inter-specific differences in isotopic trophic niches across the longitudinal estuary gradient were revealed by the presence of autotrophic sources that were isotopically distinct among the estuarine zones.

Nevertheless, the isotopic models confirmed that clupeid species feeding in the water column and mugilids feeding in the surface sediment were the main intermediate prey linking predatory fish species to the main autotrophic sources, microphytobenthos, sea grass and macro-algae. This suggested that the benthic predatory species in the Pangani estuarine system are trophically linked with both planktonic and benthic components of the estuarine and marine food web but not that of the fresh water food web. Similarly, the reliance on estuarine and marine related basal food sources of the predatory species, reduces the connectivity of terrestrial and estuarine food webs, a situation that threatens the stability of fish nursery food web structures. Susceptibility of estuarine food web structures would increase especially when the high rate of removal of meso predatory species will keep on increasing. This would result in the reduction of trophic connectivity and complexity, the indicators of low resilience ecosystem upon any disturbances. More studies and monitoring of all species caught by fishermen and the trophic relationship with other food web compartments will enhance our understanding of human impacts on the ecosystem in question and thus, improve the management strategies of estuarine and coastal ecosystems.
